# Genetic testing for homologous recombination repair (HRR) in metastatic castration-resistant prostate cancer (mCRPC): challenges and solutions

**DOI:** 10.18632/oncotarget.28015

**Published:** 2021-08-03

**Authors:** Rodney J. Scott, Anurag Mehta, Gabriel S. Macedo, Pavel S. Borisov, Ravindran Kanesvaran, Wafaa El Metnawy

**Affiliations:** ^1^Laureate Professor, Hunter Medical Research Institute, New Lambton Heights, NSW, Australia; ^2^Director, Department of Laboratory & Transfusion Services and Director Research, Rajiv Gandhi Cancer Institute, Delhi, India; ^3^Programa de Medicina Personalizada – Coordenador, Universidade Federal do Rio Grande do Sul, Porto Alegre, Rio Grande do Sul, Brazil; ^4^Oncologist Urologist, FSBI “N.N. Petrov NMRC of Oncology” of the Ministry Healthcare of the Russian Federation, St Petersburg, Russia; ^5^Deputy Head and Senior Consultant, Division of Medical Oncology, National Cancer Centre Singapore, Singapore; ^6^Professor of Molecular Pathology, Oncology Center School of Medicine, Cairo University, Giza, Egypt

**Keywords:** metastatic castration-resistant prostate cancer, homologous recombination repair, next generation sequencing, genetic testing

## Abstract

Patients with metastatic castration-resistant prostate cancer (mCRPC) have an average survival of only 13 months. Identification of novel predictive and actionable biomarkers in the homologous recombination repair (HRR) pathway in up to a quarter of patients with mCRPC has led to the approval of targeted therapies like *poly-ADP ribose polymerase inhibitors* (PARPi), with the potential to improve survival outcomes. The approval of PARPi has led to guideline bodies such as the National Comprehensive Cancer Network (NCCN) to actively recommend germline and or somatic HRR gene panel testing to identify patients who will benefit from PARPi. However, there are several challenges as genetic testing is still at an early stage especially in low- and middle-income countries, with cost and availability being major impediments. In addition, there are issues such as choice of optimal tissue for genetic testing, archival, storage, retrieval of tissue blocks, interpretation and classification of variants in the HRR pathway, and the need for pretest and pos*t*-test genetic counseling. This review provides insights into the HRR gene mutations prevalent in mCRPC and the challenges for a more widespread gene testing to identify actionable germline pathogenic variants and somatic mutations in the HRR pathway, and proposes a clinical algorithm to enhance the efficiency of the gene testing process.

## INTRODUCTION

Metastatic castration-resistant prostate cancer (mCRPC) is a latestage disease with an average survival of ≤13 months [1]. Despite significant therapeutic advances, mCRPC still remains a lethal disease. Identification of specific novel predictive biomarker mutations in mCRPC is opening up new therapeutic targets. In this context, germline or somatic mutations in genes involved in DNA damage repair (DDR) through the homologous recombination repair (HRR) pathway have been identified in 15% to 25% of mCRPC [2].

Depending on the testing strategy and the genes being evaluated, the frequency of HRR mutations varies from 11.8% for germline mutations and 23% for somatic mutations to 33% for germline and somatic mutations together [3–6]. Recently, olaparib, a poly-ADP ribose polymerase inhibitors (PARPi) has been approved for the treatment of patients with deleterious or suspected deleterious germline or somatic HRR gene-mutated mCRPC while rucaparib is approved for those with deleterious *BRCA* (germline and/or somatic) mutation-associated mCRPC for patients who have progressed (following prior treatment with enzalutamide or abiraterone) [7, 8]. Upon PARPi approval, the National Comprehensive Cancer Network (NCCN) updated guidelines (version 2, 2020) now recommend germline and/or somatic HRR gene panel and BRCA testing to identify pathogenic mutations for treatment with olaparib and rucaparib [9]. Though genetic testing for patients with mCRPC is strongly recommended, several challenges hinder its routine practice. This narrative review provides an overview of mCRPC epidemiology, elucidates the challenges in next-generation sequencing (NGS)-based gene panel testing, and proposes an algorithm for genetic testing in patients with mCRPC. Furthermore, the impact of genetic testing on personalized medicine and the regional and global challenges related to genetic counseling of individuals carrying pathogenic HRR variants are also discussed.

### Epidemiology of mCRPC

Several clinical studies have indicated that 10% to 50% of prostate cancer (PCa) cases progress to mCRPC within 3 years [10, 11]. A recent systematic literature review reported the prevalence of mCRPC as 1.6-2.1 per 100 PCa cases [12]. The incidence of mCRPC in 8 European countries and Australia was found to be 76,200 with mCRPC during 2013 to 2014 [13]. A United States (US) registry-based study forecasts the incidence of mCRPC to increase by 1.03%/year through 2025, with a rapid increase for 45–54 year (2.29%/year) and 55–69 year (1.53%/year) age groups [14]. Risk factors for mCRPC include high Gleason score (>7), higher prostate-specific antigen (PSA) values, and shorter time to PSA nadir [15].

### HRR gene mutations in mCRPC

Genetic testing by NGS is often used to identify both somatic and germline mutations in cancer patients [9]. The mutations in HRR genes commonly investigated in mCRPC include *BRCA1, BRCA2, ATM, ATR, CHK1, CHK2, DSS1, RPA1, NBSI, FANCD2, FANCA, CDK12, PALB2, BRIP1, RAD51B, RAD51C, RAD51D,* and *RAD54* [16]. The presence of pathogenic HRR mutations has been associated with an early onset of disease, aggressive tumors, higher recurrence, and poor prognosis; hence, timely identification by genetic testing becomes compelling [2–4].

Genetic aberrations of *BRCA1/2* and *ATM* genes are significantly higher (19.3%) in patients with metastatic disease compared to those with advanced localized PCa (5%) [3, 17]. In an Australian study, germline pathogenic variants of HRR genes were reported in 32.1%(18/56) patients with mCRPC [18]. Table 1 provides an overview about the prevalence of HRR mutations reported in recent studies in patients with mCRPC [5, 19–23].

**Table 1:** HRR gene alterations (deleterious somatic or germline) tested in major trials in mCRPC

Study	Genes tested	Prevalence
TOPARP-A [5]	BRCA1/2, ATM, *FANCA*, CHEK2, PALB2	33%
TOPARP-B [20]	BRCA1/2, ATM, *CDK12,PALB2*, *CHEK1*,CHEK2, ARID1A, ATRX, *FANCA*, *FANCF,FANCG, FANCI, FANCM, MSH2, NBN, RAD50, WRN*	15% (BRCA1/2), 6% (ATM), 28.3% (HRR)
PROFOUND [21]	Cohort A: *BRCA1, BRCA2,* and *ATM* Cohort B: *BRIP1, BARD1, CDK12, CHEK1, CHEK2, FANCL, PALB2, PPP2R2A, RAD51B, RAD51C, RAD51D,* and *RAD54L*	27.9% (*HRR*)
TRITON 2 [19]	*BRCA1, BRCA2, ATM, BARD1, BRIP1, CDK12, CHEK2, FANCA, NBN, PALB2, RAD51, RAD51B, RAD51C, RAD51D, RAD54*	NA
TRITON 3 [22]	*BRCA1, BRCA2, ATM*	25% (*HRR*)
GALAHAD [23]	*BRCA1*, *BRCA2*, *ATM, FANCA, PALB2, CHEK2, BRIP1,* or *HDAC2*	NA

Abbreviations: ARID1A: AT-Rich Interaction Domain 1A; *ATM*: ataxia-telangiectasia mutated; *BRCA*: breast cancer gene; *BRIP1*: BRCA1 Interacting Protein C-terminal Helicase 1; *CHEK2*: Checkpoint kinase 2; CDK12: cyclin-dependent kinase 12; *FANCA*: Fanconi anemia complementation group A; *FANCL*: Fanconi anemia complementation group L; *FANCF*: Fanconi anemia complementation group F; *FANCG*: Fanconi anemia complementation group G; *FANCI*: Fanconi anemia complementation group I; *FANCM*: Fanconi anemia complementation group M; NA: not available; *PALB2*: partner and localizer of *BRCA2*; *PPP2R2A*: protein phosphatase 2 regulatory subunit B alpha.

**Figure 1:**
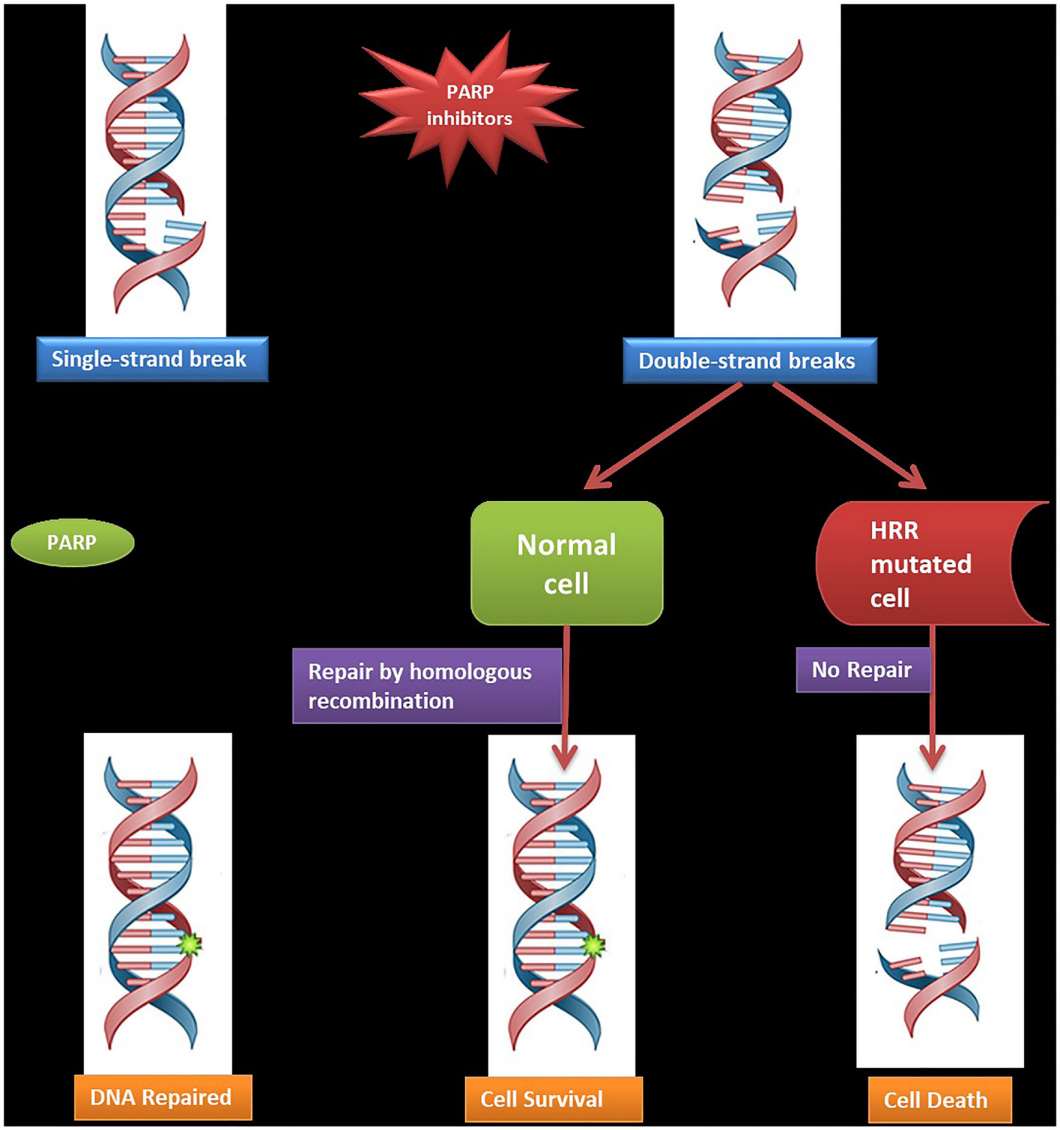
Mechanism of action of PARP inhibitors.

Pathogenic germline variants and clinically significant somatic mutations of HRR genes render cancer cells vulnerable by the principle of synthetic lethality to the action of PARPi (Figure 1) and other evolving targeted therapies, paving the way for precision medicine [5, 6]. Testing for genetic variation in HRR genes is thus important for risk stratification and making treatment decisions. Several multigene testing panels are commercially available worldwide, depending on the laboratory offerings. However, the choice of testing panel depends on factors like gene-disease validity, clinical actionability, resource availability, sample type, economic considerations, and sample logistics.

### Genetic testing in mCRPC

With the advancement of NGS technology, germline and somatic mutation testing has become more accessible and comprehensive. The NGS panel can detect different genetic aberrations, point mutations, indels, and copy number variations (CNVs) in a single test, in shorter turnaround times. In addition, NGS testing is easy to scale up to test a larger population. However, there are important questions that need to be addressed when considering genetic testing. The subsequent sections present individual challenges with possible solution(s).

#### Challenges in patient selection

Though the risk of family history is well established, prior to 2019, there were no clear guidelines specifying which patients should be considered for genetic testing. The European Association of Urology (EAU) 2019 guidelines do not have a clear recommendation for genetic testing, while the American College of Medical Genetics and Genomics (ACMG) guidelines state that germline testing should be performed if ≥3 first-degree relatives or ≥2 first-degree relatives <55 years of age are diagnosed with PCa, or patients present with a high-risk PCa (Gleason score >7), or there is a family history of 2 individuals with breast, ovarian, or pancreatic cancers [24]. The 2019 Philadelphia Prostate Cancer Consensus and the NCCN 2020 (version 2) guidelines (Table 2) also make clear recommendations on patient selection criteria for genetic testing in mPCa [9, 25].

**Table 2:** NCCN version 2; 2020 recommendations for genetic testing and treatment strategies [9]

Germline testing	Somatic testing	
Recommended	Recommended	Considered
• Strong positive family history • High-risk or very high-risk localized PCa or metastatic PCa regardless of family history • Intraductal histology • Ashkenazi Jewish ancestry	• Patients with localized or metastatic PCa • Patients with low and favorable intermediate risk PCa and life expectancy ≥10 years	NA
• Regional (Any T, N1, M0)	NA	• Regional (Any T, N1, M0) • Tumor test for HRR, MSI, and dMMR
• Metastatic (Any T, any N, M1)	• Metastatic (Any T, any N, M1) • Tumor test for HRRm	• Metastatic (Any T, any N, M1) • Tumor test for MSI or dMMR
**Recommendations for specific HRR gene testing**		
• Testing should include *BRCA1/2, ATM, PALB2,* and *CHEK2* HRR genes	• Testing should include *BRCA1/2, ATM, PALB2, CHEK2, FANCA, RAD51D,* and *CDK12* *HRR* genes • If somatic HRR mutations are identified, patients should be referred for genetic counseling	
**Treatment strategy**		
• Olaparib is a treatment option (category 1 recommendation) for patients with mCRPC and a pathogenic mutation (germline and/somatic) in a HRR gene (*BRCA1/2, ATM, BARD1, BRIP1, CDK12, CHEK1, CHEK2, FANCL, PALB2, RAD51B, RAD51C, RAD51D*, or *RAD54L*), who have been treated with androgen-receptor directed therapy. Patients with *PPP2R2A* mutations in the PROFOUND trial experienced an unacceptable risk-benefit profile. Therefore, olaparib is not recommended in patients with *PPP2R2A* mutations. • Rucaparib is a treatment option (category 2A recommendation) for mCRPC and a pathogenic *BRCA1/2* mutation (germline and/or somatic) who have been treated with androgen receptor-directed therapy and a taxane based chemotherapy. If the patient is not fit for chemotherapy, rucaparib can be considered even if taxanebased therapy has not been given.		

Abbreviations: *ATM*: ataxia-telangiectasia mutated; *BRCA*: breast cancer gene; *CHEK2*: checkpoint kinase 2; dMMR: mismatch repair; *DNA*: deoxyribonucleic acid; *FANCA*: Fanconi anemia complementation group A; MSI: microsatellite instability; NCCN: National Comprehensive Cancer Network; *PALB2*: partner and localizer of BRCA2; PCa: prostate cancer; *PPP2R2A*: Protein Phosphatase 2 Regulatory Subunit Balpha.

Age should also be considered while selecting patients for genetic testing. Patients should be considered for genetic testing for early-onset disease, diagnosis of metastatic disease, and after failure of hormonal agents or previous therapies [9]. Although germline testing is recommended for high-risk patients, this might be challenging and not always be possible given the shortage of cancer genetic clinics and clinical cancer geneticists and genetic counselors [26, 27]. This shortage is prevalent in low-and-middle income countries (LMICs) where cancer genetic testing and counseling is still in its infancy [28].

#### Challenges in gene selection: need for pragmatic testing in mCRPC

The NGS-based test generally consists of multiple genes, including several genes associated with cancer risk factors. Some Food and Drug Administration-approved NGS testing panels include the FoundationOne CDx, MSK-IMPACT, and Oncomine Dx with an ability to target 324, 468, and 23 genes, respectively [29–31]. The NGS gene panels are customizable and provide flexibility to select therapeutically actionable genes for specific testing purposes of germline and tissue testing [32]. For example, NGS panel can be customized to identify the 14 qualifying HRR pathway genes in patients with mCRPC. Several epidemiological and genome-wide assays have reported a high frequency of alterations in the *BRCA1/2* genes and *ATM* in patients with mCRPC [3]. In a consensus report on the role of genetic testing in mCRPC patients, experts agreed on the requirement for confirmatory germline *BRCA1/2* (89% agreement) and *ATM* testing (61% agreement) when tumor sequencing is positive for the same genes [33]. In the PROFOUND study, a specialized 15 predefined HRR gene assay based on FoundationOne CDx NGS assay was used [6]. In the recent PROFOUND, TRITON2, TRITON3, and GALAHAD studies, patients with deleterious somatic or germline HRR gene mutations were recruited (Table 1) [19, 21–23, 34].

NGS based testing although assisting in precision oncology has some limitations. Tumor heterogeneity either static (in the tumor tissue) or dynamic (in different time points of tumor biopsy-plasma sampling or of plasma sampling), or due to different sequencing techniques is a concern. Several NGS panels are available for testing of tumor samples. A careful consideration of the NGS panel after considering targetable mutations will lead to cost-efficient testing for mCRPC [35]. Cost-effectiveness of genetic testing is a concern, especially in the LMICs. The multigene panels include several genes, many of which are not required. Thus, targeted testing for genetic mutations in selected patients is a viable option. This is also consistent in breast and ovarian cancer where targeted testing of *BRCA* mutations in high-risk patients is conducted [36, 37]. Considering the frequency of *BRCA* and *ATM* mutations in mCRPC, targeted genetic testing for these mutations should be considered in the front-line followed by other mutation testing in case of negative results.

#### Challenges in sample selection: blood vs. tumor vs. circulating DNA (ctDNA)

**Figure 2:**
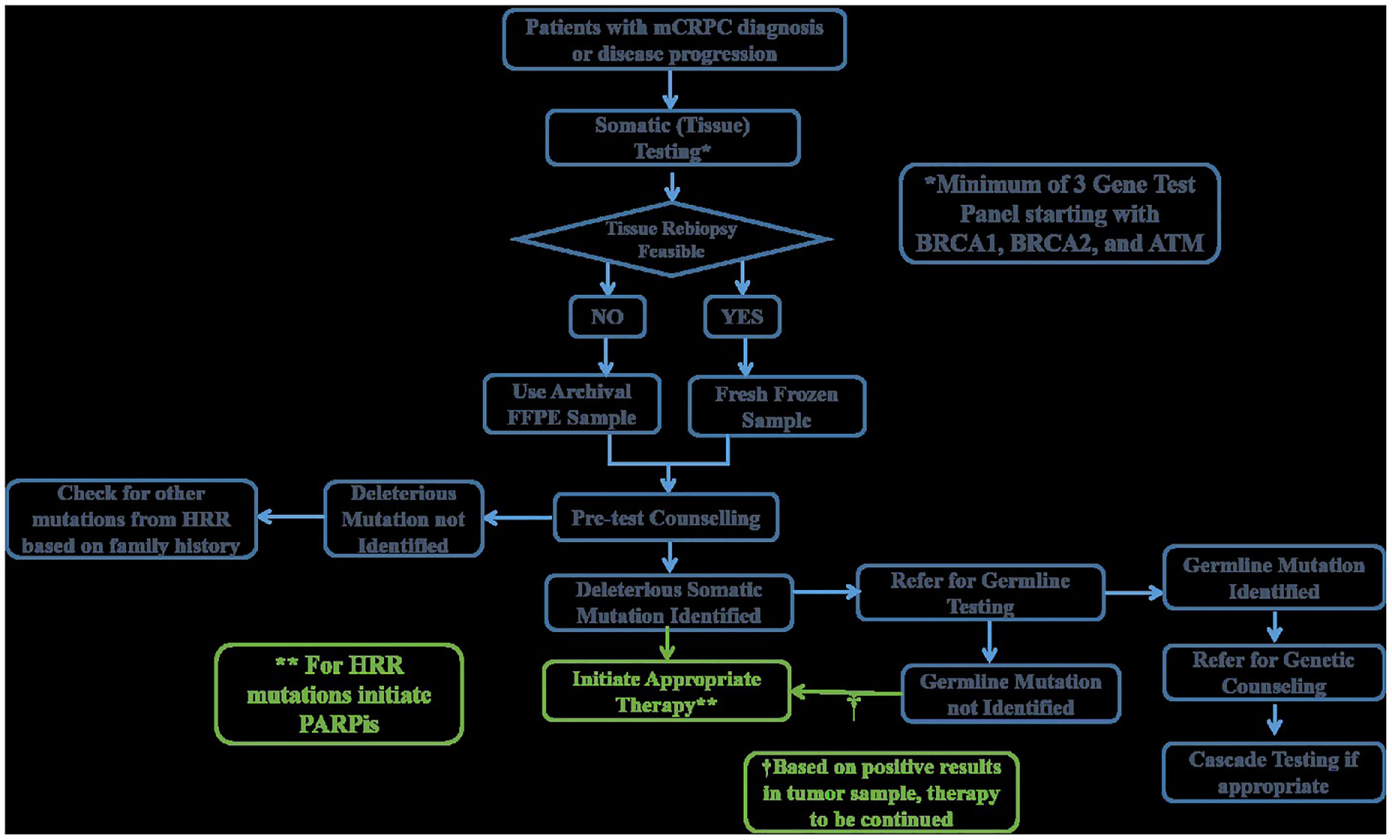
Clinical algorithm for genetic testing.

Selection of the sample for genetic testing is one of the major concerns, as viable biomaterial defines the success or failure of genetic test. Blood samples identify only germline mutations, whereas tumor or tissue samples or samples from metastatic tissues are essential for the identification of germline and somatic mutations together [38]. The prevalence of germline mutations in mCRPC is approximately 12%, while the TOPARP-A study identified 33% mutations (including germline and somatic) [3–5]. In the PROFOUND study, 27.9% (778/2792) of patients had somatic mutations in at least 1 of the 15 prespecified genes of the HRR panel [6]. Genetic testing with tumor tissue is a proven, feasible, and reliable option in a clinical setting to detect both types of mutations [36–38]. Germline testing, though well established, has a disadvantage of missing patients who possess somatic mutations that could be treated with targeted therapeutic agents. Germline testing is important to confirm the hereditary nature of the disease, be vigilant for other cancers, and recruit related unaffected individuals, who may be at an increased risk, into active surveillance programs. Germline and somatic testing use different reporting formats and provide separate interpretations. Although tumor-based testing potentially identifies both germline and somatic mutations, it is unable to differentiate them. Some laboratories conduct additional testing with paired tumor-normal specimens, which enables reporting the presence of germline variants [39]. These results can then be discussed with the patient, and after appropriate counseling, germline testing of patient as well as high-risk relatives can be started using blood or saliva samples. Somatic testing with target genes (initiated with *BRCA1/2* and *ATM* and followed by other genes) can be used as an initial screening test to provide personalized precision medicine to patients. This decreases the amount of time and resources spent on blood-based germline testing followed by tumor testing to identify a somatic mutation, in the absence of germline mutations. An algorithm for sequential testing of patients is provided in Figure 2.

#### Circulating tumor DNA

Liquid biopsy is a minimally invasive method of testing, which evaluates ctDNA in blood, and is an important method for real-time evaluation of germline variants as well as somatic mutations [38]. In patients where tumor biopsies are not feasible, ctDNA-based genetic testing is becoming popular to identify genetic alterations. The ctDNA typically represents 0.01% to 90% of the total cell-free DNA found in blood, and is usually found at a lower frequency in the plasma [40, 41]. Quantification of tumor-specific mutations from ctDNA has been validated with almost 80% concordance (compared with tumor tissue) in colorectal, lung, and breast cancer. Likewise, ctDNA testing in mCRPC is an upcoming area of interest as availability of evaluable tumor tissue and performing a biopsy remain challenging. Evaluation of ctDNA was an exploratory endpoint in TOPARP-A and PROFOUND studies [5, 6]. TRITON was the first study program to screen patients using the ctDNA plasma testing panel. The ctDNA burden was significantly higher (*P* < 0.0001) in patients who had progressed on prior androgen deprivation therapy (ADT) and taxane-based chemotherapy (TRITON2) vs. ADT alone (TRITON3). This study successfully identified mCRPC patients with HRR mutations [34]. In a study on *de novo* metastatic castration-sensitive PCa patients, those with prior ADT treatment had significantly lower ctDNA fractions than ADT-naïve patients (*P* = 0.009) [42]. Although promising, the technique involves several challenges and lacks reliability as the amount of ctDNA released in blood might vary depending on the disease stage, prior therapies, extent of metastases, amount of time sample is stored, and genes being evaluated. Thus, in mCRPC testing, ctDNA can be considered as a good complementary approach. The challenges associated with ctDNA analysis are elaborated in the subsequent sections. The advantages and disadvantages of the different gene testing strategies are summarized in Table 3 [9, 43].

**Table 3:** Comparison of gene testing strategies [9, 43]

Method of testing	Sample required	Advantages	Limitations
Germline Testing	Blood or saliva	• Germline mutations detected reliably • Large panels of tests available which can detect germline mutations in mCRPC	• Unable to detect somatic mutations relevant to treatment selection
Somatic Testing	Tumor Tissue/metastatic tissue	• Can detect germline and somatic mutations, which might be relevant for initiating targeted therapies • Provides information about translocations and amplifications • A multigene panel of tests available with testing for >300 genes possible	• Tumor heterogeneity might result in missing late somatic mutations especially if testing is conducted on archival sample • Somatic testing is less sensitive, and thus robustly validated somatic testing is required
Circulating Tumor DNA (ctDNA)	Plasma	• Can identify germline and somatic mutations relevant for targeted therapies • Minimally invasive process for sample collection as the biomaterial required is blood • Provides insight into the subclonal population that may be more relevant to current disease state	• Not enough evidence about shedding pattern of ctDNA in blood circulation in mCRPC • Availability of robustly validated HRR gene panel test • Panels may not have nonactionable genes still relevant for PCa • Chance of missing a germline variant if not sequencing the whole gene due to small size of ctDNA

Abbreviations: mCRPC: metastatic castration-resistant prostate cancer; ctDNA: circulating tumor DNA; PCa: prostate cancer.

#### Challenges in tissue processing

Tumor tissue is the gold-standard sample type for biomarker testing, as it can be used to identify germline and somatic mutations. However, isolation of evaluable quantity and quality of nucleic acid from tissue samples may be an issue due to improper tissue processing or storage of formalin-fixed paraffin-embedded (FFPE) blocks. Molecular pathologists can provide information at the molecular level, in the shortest possible time, and with high reliability, enabling the oncologists to make therapeutic decisions. The three main phases of sample flow include pre-analytical (sample collection, transport to the laboratory, and sample processing), analytical (laboratory test), and post-analytical (data analysis, interpretation of results, archival of sample for further use) [44].

#### Challenges in pre-analytical and analytical phases

In the PROFOUND study, FFPE tumor tissue samples were used for genetic testing. Of the 4047 samples available, the reasons for test failure in 31% of samples were pathology review failure (6.8%), DNA extraction failure (13.2%), and failure after DNA extraction (6.9%); 4.1% of patients failed in more than 1 category. Samples were mainly derived from the archived primary tumor tissue (89.9%). Though a higher prevalence of HRR mutations was obtained from the metastatic tissue samples, less than 5% of metastatic samples were from bone tissue, emphasizing inaccessibility of bone metastatic tissue [6].

One of the critical challenges during the pre-analytical phase is obtaining an appropriate and adequate tumor sample by biopsy. The quality of samples varies depending on the biopsy route (trans-rectal or trans-perineal route with trans-rectal ultrasound guided) and the technique (fine-needle aspiration or core needle). Although a higher prevalence of mutations is observed in metastatic tissue, obtaining a sample from a metastatic site is difficult in mCRPC as the most frequent site of metastasis is bone. Isolation of DNA from bone requires modified isolation protocols involving a decalcification process, which can have a negative impact on the quality of the DNA. When bone metastasis samples are to be used for genetic testing, decalcification should preferably be performed using ethylenediaminetetraacetic acid (EDTA) as EDTA does not degrade DNA integrity [45]. Heterogeneity within the tumor tissue is also a limitation, as the sample obtained may not be completely representative of the tumor biology, and hence may not represent the entire genomic mutation profile of the tumor. Thus, amplification of genes through PCR or isothermal DNA amplification-based processes is required to get adequate samples for mutation detection. However, these processes can cause a representation bias [46].

Improper fixation of tumor samples poses specific challenges for the integrity of DNA. Fresh frozen samples are a feasible sample type for genetic analysis. However, in clinical settings, it may not always be possible to perform a re-biopsy, and determination of tumor content may also be a challenge before proceeding for NGS-based HRR gene testing. In such cases, archived samples are often used, with FFPE samples being the most preferred option [47–49]. The tumor content evaluation of FFPE samples is critical to identify successful genetic alterations through NGS gene panel testing. If tumor content is inadequate, it is advisable to obtain micro-dissected target tissue by a trained pathologist to enrich the tumor content [50]. To ensure good quality FFPE samples, it is recommended that tissue fixation be performed with 10% neutral buffered formalin for 1 day (8–24 hours), with time to fixation being not more than 20 minutes, and heat treatment of tissue lysates at 95°C for 30 minutes. The thickness of the section should be 5–10 μm. FFPE sections for DNA extraction should be derived from a single representative block per case and contain 30–50 μm depth tissue sections containing at least 100 ng of DNA corresponding to 15,000 cells.

Another challenge is the yield from archived vs. fresh samples. In a systematic analysis of tissue samples, the effect of storage period was evaluated on the quantity/quality of the extracted nucleic acids and proteins from the FFPE blocks of malignant tumors of lung, thyroid, and salivary gland stored over several years. No significant difference was found between macromolecules extracted from blocks stored over 11–12 years, 5–7 years, or 1–2 years in comparison with blocks from the current year [51]. Although the study did not report any significant difference, the quality of the FFPE samples and the storage conditions did affect the overall quality of the blocks. Extraction and analysis of nucleic acids requires specialty laboratories where stringent processes need to be followed to avoid cross-contamination and where quantification of samples is performed with validated instruments providing high specificity and accuracy [52].

The ctDNA shed in blood can provide a comprehensive view of tumor heterogeneity. The pre-analytical challenges associated with ctDNA testing include: low yield during initial phases and high yield but interpersonal variations in metastatic stage [53]. Hence, the actual amount of ctDNA may vary. Another important consideration is the detection of large gene rearrangements like those found in *BRCA1/2* genes and *ATM*. The usual size of circulating DNA fragments in blood ranges from 185 to 200 base pairs. Larger gene deletions or duplications may not be efficiently screened by ctDNA testing [54]. The concentration of ctDNA is also affected by other pre-analytical factors like blood collection tubes, sample type (plasma preferred to serum), and processing time. The time interval between collection and centrifugation is critical. Delay in processing (> 4 hours) can cause dilution of tumor DNA by normal DNA due to leukocyte lysis. Blood collection tubes with nucleic acid stabilization fluid that prevent cross-linking (Streck tubes) are preferred for sample storage conditions if sample processing time is more than 4 hours. Proper centrifugation at low speed followed by high speed is important for isolation of ctDNA. Currently, ctDNA is used mainly in a research setting and its applicability in real-world genetic testing needs to be further evaluated [55].

#### Challenges in post-analytical phase

One major post-analytical challenge is the ability to detect genetic alterations in the archived and fresh FFPE tissue samples. In mCRPC, archived FFPE blocks obtained at initial diagnosis are preferred as re-biopsy may not be feasible for most patients. In addition, metastasis occurs in bone, which is not an ideal sample for genetic testing. Improper storage of archived FFPE tissue blocks results in a lower yield of quality DNA to proceed for NGS test [49]. Since the time gap between initial PCa diagnosis and mCRPC setting may be several years, retrieval of the archived FFPE tissue blocks may pose a critical challenge. Many regulatory authorities recommend the FFPE tissue blocks to be stored for 5–10 years [56, 57]. The National Accreditation Board For Testing & Calibration Laboratories (NABL-India) and College of American Pathologists (CAP) guidelines recommend storage of FFPE tissue blocks for 10 years. However, countries like Brazil and Japan do not have any set guidelines regarding the storage timelines. Thus, long-term storage and retrieval of FFPE tissue blocks is critical for successful genetic testing in mCRPC setting.

Reporting of genetic test results should follow the ACMG guidelines for germline mutations and A Joint Consensus Recommendation of the Association for Molecular Pathology (AMP), American Society of Clinical Oncology (ASCO), and CAP for somatic variants [58].

In general, test reports must include the following: patient demographic data; classification of results into pathogenic/likely pathogenic or likely non-pathogenic/non-pathogenic (germline), strong-clinical significance/potential clinical significance or likely benign/benign (somatic); and variant of uncertain significance (VUS) when interpreting results regarding the functional and therapeutic impact of the genetic variants, following Human Genome Variation Society (HGVS) nomenclature for variant calling [39, 59]. To facilitate the increasing information on genome sequencing and genetic mutations, several global public databases and cancer-specific databases have been established, which can be used to identify genetic variants and guide management. Other sources for identifying whether a mutation is pathogenic, benign, or VUS include data repositories, such as reference sequence information, population databases, and germline variant databases. These databases help in identifying causal relationship between genetic variant and patient health status [39, 60]. These tools should be used during development of the report for including information on pathogenicity of the mutations.

**Figure 3:**
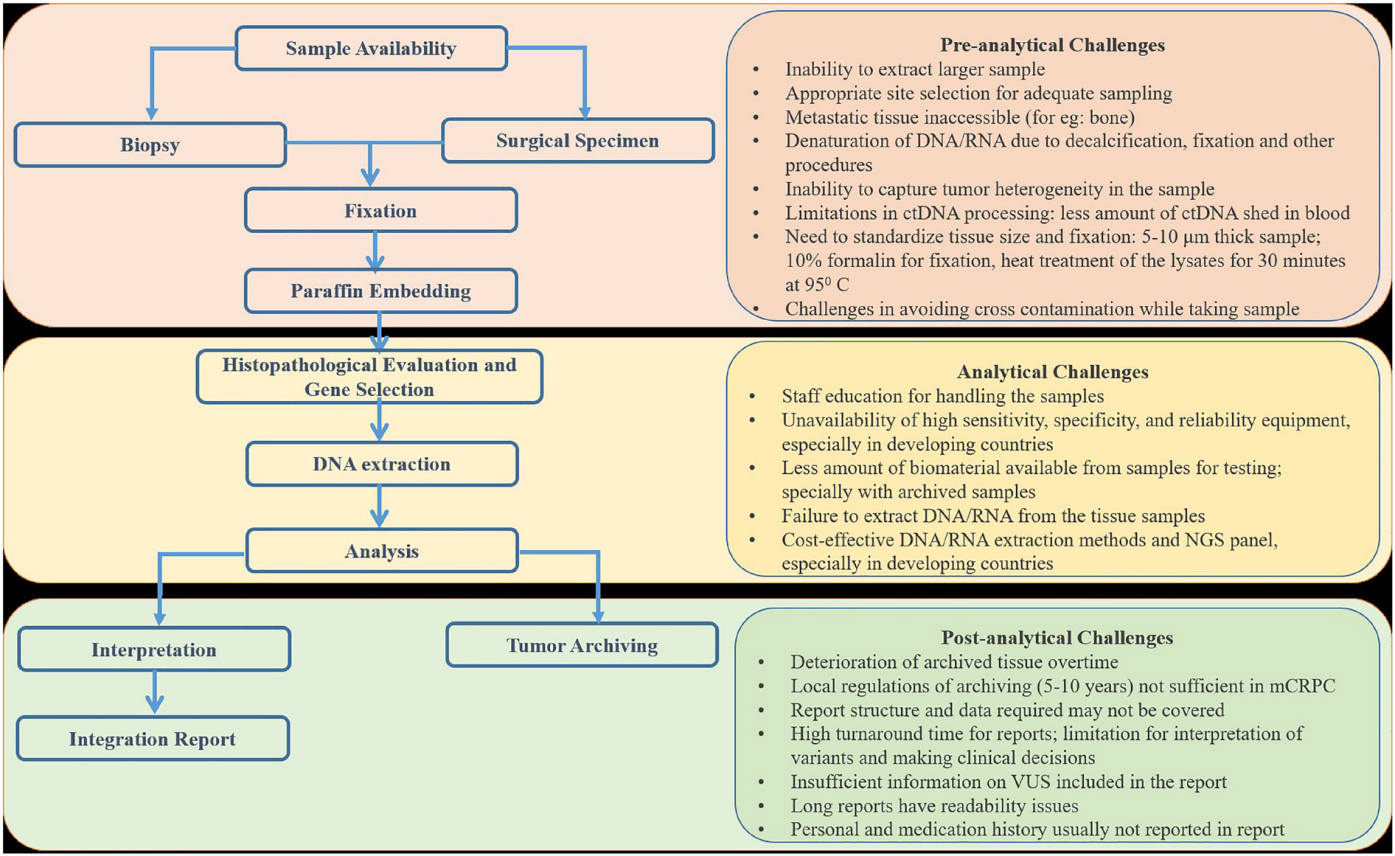
Flow of sample during genetic testing and challenges associated with each stage in LMICs.

Additionally, reports must include the percentage of neoplastic cell content, test methodology, test limitations, and genetic counseling recommendations. This can facilitate appropriate communication to the patient by the treating physician and inclusion of genetic counselors in to the multidisciplinary team, to streamline specific treatment strategies [52]. Figure 3 provides a brief overview of the sample flow for molecular testing and the pre- and post-analytical challenges associated at each stage, especially in LMICs.

#### Genetic counseling

As the role of genetic evaluation with NGS testing in mCRPC treatment increases, the need for genetic counseling will also increase. Genetic counseling for ovarian and breast cancer patients is well established as the role of genetics in these cancers was revealed over two decades ago. However, genetic counseling in mCRPC is in a nascent stage. Traditionally, genetic counseling is conducted by in-person visits in a dedicated genetic clinic, but other methodologies like telehealth discussions, online counseling, and group discussions are required to overcome geographic barriers [61]. Along with post-test genetic counseling, pre-test genetic counseling is an important aspect as it helps patients prepare for the genetic tests, and allows them to make informed decisions about the same [62]. However, in many countries, having dedicated genetic clinics is considered unmanageable because of the time and resources required; an alternate practical approach involves tumor testing to identify pathogenic variants and providing counseling before germline testing in the case of positive results for a somatic mutation [63]. This alleviates the pressures involved in up-front counseling, in case of insufficient counselors or clinical geneticists (especially in countries with limited resources) and ensures it is provided to only those who require it [63].

### Clinical implication of genetic testing

Over the past few years, the treatment for mCRPC has seen an exponential evolution, with many chemotherapeutic drugs and hormonal treatments being approved. With the advent of new discoveries in the field of biomarkers and genetic testing for mCRPC, precision medicine for patients with a positive mutation status is now within reach. Patients with somatic or germline HRR mutations become eligible for treatment with targeted agents like PARPi. Additionally, there are larger implications such as implementation of risk reduction strategies for second cancers, testing of first- and second-degree family members to identify mutation carriers, and active surveillance of family members to allow screening for the high-risk of related cancers.

#### Therapeutic implications for patients carrying HRR gene mutations

Recently, PARPi have achieved significant response rates in mCRPC patients harboring HRR mutations including *BRCA1/2* (Table 4) [5, 6, 19, 20, 23, 64]. Though both olaparib and rucaparib have been approved for treatment of patients with mCRPC, there are differences in the approval based on the genetic mutations as well as the approval differing across countries (Table 5) [7, 8, 65–67]. Also, though olaparib is approved for patients with HRR mutations, the highest benefit was observed in patients with *BRCA1/2* mutated tumors.

**Table 4:** Clinical studies of PARP inhibitors in mCRPC with HRR mutations

Study	Agent	Patients Enrolled	Key Findings
TOPARP-A [5]	Olaparib	Olaparib 49	**Overall RR:** 33% (16/49) **RR in HRR positive subgroup**: 88% (14/16) **PFS:** HRR+ve: 9.8 vs. HRR-ve: 2.7 months; *P* < 0.001 **OS:** HRR+ve: 13.8 vs. HRR-ve: 7.5 months; *P* = 0.05
TOPARP-B [20]	Olaparib 400 mg vs. Olaparib 300 mg (randomized 1:1)	Olaparib 400: 49 Olaparib 300: 49	**RR:** Olaparib 400 mg group: 54.3% vs. olaparib 300 mg group: 39.1% **PFS:** Olaparib 400 mg 5.5 months vs. olaparib 300 mg 5.4 months **OS:** Olaparib 400 mg 14.3 vs. olaparib 300 mg 10.1 months
PROFOUND [6]	Olaparib 300 mg vs. enzalutamide or Abi (pcNHA; randomized 2:1)	Cohort A+B: Olaparib: 256 vs. pcNHA: 131 Cohort A: Olaparib: 162 vs. pcNHA: 83	**Cohort A+B** **RR:** olaparib 22.0% vs. ADT 4.0% **PFS:** olaparib 5.8 vs. ADT 3.5 months **OS:** olaparib 17.5 vs. ADT 14.3 months **Cohort A** **RR:** olaparib 33.0% vs. ADT 2.0% **PFS:** olaparib 7.4 vs. ADT 3.6 months **OS:** olaparib 18.5 vs. ADT 15.1 months
TRITON2 (preliminary results) [19]	Rucaparib 600 mg	136	**RR:** 44% in patients with *BRCA1/2* mutations Confirmed PSA response in 51.1% patients in BRCA1/2 group, 1 patient with a *CDK12* alteration, 1 patient with a *BRIP1* alteration, and 1 patient with a *FANCA* alteration
GALAHAD (preliminary results) [23]	Niraparib 300 mg	Total: 81; BRCA ½: 46 non-BRCA: 35	**RR:** *BRCA ½* 41% vs. *Non-BRCA* 9% **PFS:** *BRCA ½* 8.2 vs. *Non-BRCA* 5.3 months **OS:** *BRCA ½* 12.6 vs. *Non-BRCA* 14
Clarke et al. 2018 [64]	Abi with Olaparib 300 mg or placebo (randomized 1:1)	Abi+Olaparib: 71 Abi+placebo: 71	**RR:** Abi+Olaparib 27% vs. Abi+placebo 32% **PFS:** Abi+Olaparib 13.8 vs. Abi+placebo 8.2 months **OS:** Abi+Olaparib 22.7 Abi+placebo 20.9 months

Abbreviations: Abi: abiraterone; HRR: homologous recombination repair; OS: overall survival; pcNHA: physician choice novel hormonal agent (abiraterone or enzalutamide); PFS: progression-free survival; RR: response rate.

**Table 5:** Approval status of olaparib and rucaparib for prostate cancer

**Indications for Olaparib** United States: Treatment for adult patients with deleterious or suspected deleterious germline or somatic homologous recombination repair (HRR) gene-mutated CRPC who have progressed following treatment with enzalutamide or abiraterone. United Kingdom: As monotherapy for the treatment of adult patients with metastatic castration-resistant prostate cancer (mCRPC) and *BRCA1/2*-mutations (germline and/or somatic) who have progressed following prior therapy that included a new hormonal agent. Canada: As monotherapy for the treatment of adult patients with deleterious or suspected deleterious germline and/or somatic *BRCA* or *ATM* mutated mCRPC who have progressed following prior treatment with a new hormonal agent. *BRCA* or *ATM* mutations must be confirmed before olaparib treatment is initiated. India: Treatment of patients with mCRPC and HRR gene mutations (germline and/or somatic) who have progressed following a prior new hormonal agent.
**Indications for Rucaparib** United States: Treatment of adult patients with deleterious BRCA mutation (germline and/or somatic)-associated mCRPC who have been treated with androgen receptor directed therapy and a taxane-based chemotherapy. This indication is approved under accelerated approval based on objective response rate and duration of response.

Abbreviations: *BRCA*: breast cancer gene; HRR: homologous recombination repair; mCPRC: metastatic castrate-resistant prostate cancer.

#### Implications if tested negative for mutations/VUS

When mCRPC patients test negative for clinically significant somatic or pathogenic germline mutations, the results are interpreted as inconclusive for therapeutic implications since there may be other genetic factors that are not tested [68]. Genetic tests also include the reporting of VUS, which are reported in almost 30% of cases [69]. Clinical decisions should not be based on a VUS report. Caregivers, patients, and family members should wait for the reclassification of the VUS, as it is an ongoing process and may be reclassified to benign or pathogenic [70]. Global public and cancer specific databases can be useful in identification of genetic variants and are also useful in management [39, 60].

## CONCLUSIONS

mCRPC management has significantly advanced over the past 5 years owing to the continuously evolving knowledge on actionable genetic mutations. However, in countries with limited resources, HRR testing in mCRPC is still at its inception, and patients may not opt to test because of a lack of reimbursement by way of insurance. In addition, on most occasions, the tissue available for genetic testing is from archival samples, which poses multiple challenges. Thus, testing for the three predominant genes (*BRCA1/2* and *ATM*) as an initial step and then proceeding by an elimination method can improve the efficiency of genetic testing in mCRPC in countries with limitations on genetic testing resources. While ctDNA is a promising approach for genetic testing, it has several limitations, such as inavailability of robust testing strategies for PCa and lack of knowledge about the DNA shredding pattern in PCa. We have proposed an algorithm for genetic testing of HRR mutations in LMICs considering the prevalence of mutations in mCRPC, which focuses on testing for *BRCA1/2* and *ATM* mutations initially followed by other HRR mutations in case *BRCA1/2* and *ATM* are negative. Along with the algorithm, we have proposed recommendations for HRR testing strategy in mCRPC (Table 6). Recent studies on patients with mCRPC and HRR mutations have reported significant response and improvement in progression-free survival and overall survival with PARPi. Thus, inclusion of genetic testing and counseling for these mutations will be critical for improving patient outcomes in mCRPC.

**Table 6:** Recommendations for HRR testing in mCRPC

• Obtain an appropriate and adequate tumor sample • Although fresh samples are recommended, FFPE tissue blocks stored in appropriate storage conditions as per CAP guidelines can be utilized for genetic testing • Comprehensive genomic profiling of mCRPC for HRR genes at presentation • NGS-based test to identify deleterious/suspected deleterious somatic mutations, and if identified, proceed for qualifying germline HRR mutations identification • Prioritize *BRCA1/2* and *ATM* (somatic and or germline) testing in the HRR pathway • Reporting of germline results should follow ACMG guidelines and somatic results should follow A Joint Consensus Recommendation of the AMP, ASCO, and CAP • Incorporate pre-test and post-test genetic counseling

Abbreviations: ACMG: American College of Medical Genetics; AMP: Association for Molecular Pathology; ASCO: American Society of Clinical Oncology; *ATM*: ataxia-telangiectasia mutated; *BRCA*: breast cancer gene; CAP: College of American Pathologists; FFPE: formalin-fixed paraffin embedded; HRR: homologous recombination repair; NGS: next generation sequencing; mCPRC: metastatic castrate-resistant prostate cancer.

## Abbreviations

ACMG: American College of Medical Genetics
AMP: Association for Molecular Pathology
ARID1A: AT-Rich Interaction Domain 1A
ASCO: American Society of Clinical Oncology
ATM: ataxia-telangiectasia mutated
BRCA: breast cancer gene
BRIP1: BRCA1 Interacting Protein C-terminal Helicase 1
CAP: College of American Pathologists
CDK12: cyclin-dependent kinase 12
CHEK2: Checkpoint kinase 2
ctDNA: circulating tumor DNA
dMMR: mismatch repair
DNA: deoxyribonucleic acid
FANCA: Fanconi anemia complementation group A
FANCF: Fanconi anemia complementation group F
FANCG: Fanconi anemia complementation group G
FANCL: Fanconi anemia complementation group L
FFPE: formalin fixed paraffin embeddded
HRR: homologous recombination repair
mCRPC: metastatic castration-resistant prostate cancer
MSI: microsatellite instability
NCCN: National Comprehensive Cancer Network
NGS: next generation sequencing
OS: overall survival
PALB2: partner and localizer of BRCA2
PARPi: poly (ADP-ribose) polymerase inhibitors
PCa: prostate cancer
PCR: polymerase chain reaction
PFS: progression-free survival
PPP2R2A: protein phosphatase 2 regulatory subunit B alpha
VUS: variants of unknown significance

## References

[1] Moreira DM , Howard LE , Sourbeer KN , Amarasekara HS , Chow LC , Cockrell DC , Pratson CL , Hanyok BT , Aronson WJ , Kane CJ , Terris MK , Amling CL , Cooperberg MR , Freedland SJ . Predicting Time From Metastasis to Overall Survival in Castration-Resistant Prostate Cancer: Results From SEARCH. Clin Genitourin Cancer. 2017; 15:60–66.e2. 10.1016/j.clgc.2016.08.018. 27692812 PMC5536956

[2] Furlow B . NCCN: More Genetic Testing to Inform Prostate Cancer Management. Cancer Network. 2019. https://www.cancernetwork.com/nccn/nccn-more-genetic-testing-inform-prostate-cancer-management.

[3] Robinson D , Van Allen EM , Wu YM , Schultz N , Lonigro RJ , Mosquera JM , Montgomery B , Taplin ME , Pritchard CC , Attard G , Beltran H , Abida W , Bradley RK , et al. Integrative Clinical Genomics of Advanced Prostate Cancer. Cell. 2015; 162:454. 10.1016/j.cell.2015.06.053. 28843286

[4] Pritchard CC , Mateo J , Walsh MF , De Sarkar N , Abida W , Beltran H , Garofalo A , Gulati R , Carreira S , Eeles R , Elemento O , Rubin MA , Robinson D , et al. Inherited DNA-Repair Gene Mutations in Men with Metastatic Prostate Cancer. N Engl J Med. 2016; 375:443–53. 10.1056/NEJMoa1603144. 27433846 PMC4986616

[5] Mateo J , Carreira S , Sandhu S , Miranda S , Mossop H , Perez-Lopez R , Nava Rodrigues D , Robinson D , Omlin A , Tunariu N , Boysen G , Porta N , Flohr P , et al. DNA-Repair Defects and Olaparib in Metastatic Prostate Cancer. N Engl J Med. 2015; 373:1697–708. 10.1056/NEJMoa1506859. 26510020 PMC5228595

[6] de Bono J , Mateo J , Fizazi K , Saad F , Shore N , Sandhu S , Chi KN , Sartor O , Agarwal N , Olmos D , Thiery-Vuillemin A , Twardowski P , Mehra N , et al. Olaparib for Metastatic Castration-Resistant Prostate Cancer. N Engl J Med. 2020; 382:2091–102. 10.1056/NEJMoa1911440. 32343890

[7] Olaparib tablets, for oral use. USPI. 2014. https://www.azpicentral.com/lynparza_tb/lynparza_tb.pdf%23page=1.

[8] Rucaparib tablets, for oral use. USPI. 2016. https://clovisoncology.com/pdfs/RubracaUSPI.pdf.

[9] Prostate Cancer NCCN Guidelines Version 2.2020. https://www.nccn.org/professionals/physician_gls/default.aspx.

[10] Harris WP , Mostaghel EA , Nelson PS , Montgomery B . Androgen deprivation therapy: progress in understanding mechanisms of resistance and optimizing androgen depletion. Nat Clin Pract Urol. 2009; 6:76–85. 10.1038/ncpuro1296. 19198621 PMC2981403

[11] Lorente D , Mateo J , Perez-Lopez R , de Bono JS , Attard G . Sequencing of agents in castration-resistant prostate cancer. Lancet Oncol. 2015; 16:e279–92. 10.1016/S1470-2045(15)70033-1. 26065613

[12] Shore ND , Oliver L , Shui I . Review of the real-world prevalence of mHSPC, nmCRPC, mCRPC, and gene alterations associated with HRR in prostate cancer (PC). J Clin Oncol. 2020; 38:229. 10.1200/JCO.2020.38.6_suppl.229.

[13] Marteau F , Gimonet G , Gabriel S , Dinet J , Flinois A , LE Cleac’h JY . Epidemiology of Patients with Metastatic Castrate Resistant Prostate Cancer in Europe and Australia. Value Health. 2014; 17:A619. 10.1016/j.jval.2014.08.2188. 27202173

[14] Kelly SP , Anderson WF , Rosenberg PS , Cook MB . Past, Current, and Future Incidence Rates and Burden of Metastatic Prostate Cancer in the United States. Eur Urol Focus. 2018; 4:121–27. 10.1016/j.euf.2017.10.014. 29162421 PMC6217835

[15] Lin TT , Chen YH , Wu YP , Chen SZ , Li XD , Lin YZ , Chen SH , Zheng QS , Wei Y , Xu N , Xue XY . Risk factors for progression to castration-resistant prostate cancer in metastatic prostate cancer patients. J Cancer. 2019; 10:5608–13. 10.7150/jca.30731. 31632505 PMC6775699

[16] Brönimann S , Lemberger U , Bruchbacher A , Shariat SF , Hassler MR . Poly(ADP-ribose) polymerase inhibitors in prostate and urothelial cancer. Curr Opin Urol. 2020; 30:519–26. 10.1097/MOU.0000000000000776. 32427631

[17] Kote-Jarai Z , Leongamornlert D , Saunders E , Tymrakiewicz M , Castro E , Mahmud N , Guy M , Edwards S , O’Brien L , Sawyer E , Hall A , Wilkinson R , Dadaev T , et al, and UKGPCS Collaborators. BRCA2 is a moderate penetrance gene contributing to young-onset prostate cancer: implications for genetic testing in prostate cancer patients. Br J Cancer. 2011; 105:1230–34. 10.1038/bjc.2011.383. 21952622 PMC3208504

[18] Banks P , Leong HS , Ryland G . 154 DNA repair gene defects in Australian men with metastatic castration-resistant prostate cancer (mCRPC). Poster Abstracts. Asia Pac J Clin Oncol. 2017; 13:40–64. 10.1111/ajco.12719.

[19] Abida W , Bryce AH , Vogelzang NJ , Amato RJ , Percent I , Shapiro JD , McDermott R , Hussain A , Patnaik A , Petrylak D , Ryan CJ , Stanton T , Zhang J , et al. Preliminary results from TRITON2: A phase II study of rucaparib in patients (pts) with metastatic castration-resistant prostate cancer (mCRPC) associated with homologous recombination repair (HRR) gene alterations. 2018; 29:VIII272. 10.1093/annonc/mdy284.002.

[20] Mateo J , Porta N , Bianchini D , McGovern U , Elliott T , Jones R , Syndikus I , Ralph C , Jain S , Varughese M , Parikh O , Crabb S , Robinson A , et al. Olaparib in patients with metastatic castration-resistant prostate cancer with DNA repair gene aberrations (TOPARP-B): a multicentre, open-label, randomised, phase 2 trial. Lancet Oncol. 2020; 21:162–74. 10.1016/S1470-2045(19)30684-9. 31806540 PMC6941219

[21] de Bono JS , Fizazi K , Saad F , Shore N , Sandhu SK , Mehra N , Kolinsky M , Roubaud G , Ӧzguroǧlu, Matsubara N , Gedye C , Choi YD , Padua C , et al. Central, prospective detection of homologous recombination repair gene mutations (HRRm) in tumour tissue from >4000 men with metastatic castration-resistant prostate cancer (mCRPC) screened for the PROfound study. Annals of Oncology. 2019; 30:v328–29. 10.1093/annonc/mdz248.004.

[22] Ryan CJ , Abida W , Bryce AH , Balar AV , Dumbadze I , Given RW , Morris D , Petrylak DP , Redfern CH , Scher HI , Watkins SP , Simmons A , Passler L , et al. TRITON3: An international, randomized, open-label, phase III study of the PARP inhibitor rucaparib vs. physician’s choice of therapy for patients with metastatic castration-resistant prostate cancer (mCRPC) associated with homologous recombination deficiency (HRD). Journal of Clinical Oncology. 2018; 36:TPS389. 10.1200/JCO.2018.36.6_suppl.TPS389.

[23] Smith MR , Sandhu SK , Kelly WK , Scher HI , Efstathiou E , Lara PN , Yu EY , George DJ , Chi KN , Saad F , Summa J , Freedman JM , Mason GE , et al. Pre-specified interim analysis of GALAHAD: A phase II study of niraparib in patients (pts) with metastatic castration-resistant prostate cancer (mCRPC) and biallelic DNA-repair gene defects (DRD). Annals of Oncology. 2019; 30:v884–85. 10.1093/annonc/mdz394.043.

[24] Hampel H , Bennett RL , Buchanan A , Pearlman R , Wiesner GL , and Guideline Development Group, and American College of Medical Genetics and Genomics Professional Practice and Guidelines Committee and National Society of Genetic Counselors Practice Guidelines Committee. A practice guideline from the American College of Medical Genetics and Genomics and the National Society of Genetic Counselors: referral indications for cancer predisposition assessment. Genet Med. 2015; 17:70–87. 10.1038/gim.2014.147. 25394175

[25] Giri VN , Knudsen KE , Kelly WK , Cheng HH , Cooney KA , Cookson MS , Dahut W , Weissman S , Soule HR , Petrylak DP , Dicker AP , AlDubayan SH , Toland AE , et al. Implementation of Germline Testing for Prostate Cancer: Philadelphia Prostate Cancer Consensus Conference 2019. J Clin Oncol. 2020; 38:2798–811. 10.1200/JCO.20.00046. 32516092 PMC7430215

[26] Maiese DR , Keehn A , Lyon M , Flannery D , Watson M , and Working Groups of the National Coordinating Center for Seven Regional Genetics Service Collaboratives. Current conditions in medical genetics practice. Genet Med. 2019; 21:1874–77. 10.1038/s41436-018-0417-6. 30686822 PMC6752678

[27] Stoll K , Kubendran S , Cohen SA . The past, present and future of service delivery in genetic counseling: Keeping up in the era of precision medicine. Am J Med Genet C Semin Med Genet. 2018; 178:24–37. 10.1002/ajmg.c.31602. 29512888

[28] Nakamura S , Kwong A , Kim SW , Iau P , Patmasiriwat P , Dofitas R , Aryandono T , Hu Z , Huang CS , Ginsburg O , Rashid MU , Sarin R , Teo SH . Current Status of the Management of Hereditary Breast and Ovarian Cancer in Asia: First Report by the Asian BRCA Consortium. Public Health Genomics. 2016; 19:53–60. 10.1159/000441714. 26575363

[29] FoundationOne CDx™ Technical Information. 2020. https://assets.ctfassets.net/vhribv12lmne/6Rt6csmCPuaguuqmgi2iY8/e3a9b0456ed71a55d2e4480374695d95/FoundationOne_CDx.pdf.

[30] MSK-IMPACT: A Targeted Test for Mutations in Both Rare and Common Cancers. 2020. https://www.mskcc.org/msk-impact#:~:text=MSK%2DIMPACT%E2%84%A2%20stands%20for,both%20rare%20and%20common%20cancers.

[31] ThermoFisher. A new paradigm in testing for NSCLC-targeted therapies. 2020. https://assets.thermofisher.com/TFS-Assets/LSG/brochures/oncomine-dx-target-test-flyer.pdf.

[32] Damodaran S , Berger MF , Roychowdhury S . Clinical tumor sequencing: opportunities and challenges for precision cancer medicine. Am Soc Clin Oncol Educ Book. 2015:e175–82. 10.14694/EdBook_AM.2015.35.e175. 25993170 PMC5557651

[33] Giri VN , Knudsen KE , Kelly WK , Abida W , Andriole GL , Bangma CH , Bekelman JE , Benson MC , Blanco A , Burnett A , Catalona WJ , Cooney KA , Cooperberg M , et al. Role of Genetic Testing for Inherited Prostate Cancer Risk: Philadelphia Prostate Cancer Consensus Conference 2017. J Clin Oncol. 2018; 36:414–24. 10.1200/JCO.2017.74.1173. 29236593 PMC6075860

[34] Chowdhury S , McDermott R , Piulats JM , Shapiro JD , Mejlholm I , Morris D , Ostler P , Hussain A , Dumbadze I , Goldfischer ER , Pintus E , Benjelloun A , Gross ME , et al. Genomic profiling of circulating tumour DNA (ctDNA) and tumour tissue for the evaluation of rucaparib in metastatic castration-resistant prostate cancer (mCRPC). Annals of Oncology. 2018; 29:viii273. 10.1093/annonc/mdy284.004.

[35] Colomer R , Mondejar R , Romero-Laorden N , Alfranca A , Sanchez-Madrid F , Quintela-Fandino M . When should we order a next generation sequencing test in a patient with cancer? EClinicalMedicine. 2020; 25:100487. 10.1016/j.eclinm.2020.100487. 32775973 PMC7397394

[36] D’Andrea E , Marzuillo C , Pelone F , De Vito C , Villari P . Genetic testing and economic evaluations: a systematic review of the literature. Epidemiol Prev. 2015; 39:45–50. 26499415

[37] Manchanda R , Legood R , Burnell M , McGuire A , Raikou M , Loggenberg K , Wardle J , Sanderson S , Gessler S , Side L , Balogun N , Desai R , Kumar A , et al. Cost-effectiveness of population screening for BRCA mutations in Ashkenazi jewish women compared with family history-based testing. J Natl Cancer Inst. 2014; 107:380. 10.1093/jnci/dju380. 25435542 PMC4301704

[38] Cheng H , Powers J , Schaffer K , Sartor O . Practical Methods for Integrating Genetic Testing Into Clinical Practice for Advanced Prostate Cancer. Am Soc Clin Oncol Educ Book. 2018; 38:372–81. 10.1200/EDBK_205441. 30231311

[39] Li MM , Datto M , Duncavage EJ , Kulkarni S , Lindeman NI , Roy S , Tsimberidou AM , Vnencak-Jones CL , Wolff DJ , Younes A , Nikiforova MN . Standards and Guidelines for the Interpretation and Reporting of Sequence Variants in Cancer: A Joint Consensus Recommendation of the Association for Molecular Pathology, American Society of Clinical Oncology, and College of American Pathologists. J Mol Diagn. 2017; 19:4–23. 10.1016/j.jmoldx.2016.10.002. 27993330 PMC5707196

[40] Wan JCM , Massie C , Garcia-Corbacho J , Mouliere F , Brenton JD , Caldas C , Pacey S , Baird R , Rosenfeld N . Liquid biopsies come of age: towards implementation of circulating tumour DNA. Nat Rev Cancer. 2017; 17:223–38. 10.1038/nrc.2017.7. 28233803

[41] Lanman RB , Mortimer SA , Zill OA , Sebisanovic D , Lopez R , Blau S , Collisson EA , Divers SG , Hoon DS , Kopetz ES , Lee J , Nikolinakos PG , Baca AM , et al. Analytical and Clinical Validation of a Digital Sequencing Panel for Quantitative, Highly Accurate Evaluation of Cell-Free Circulating Tumor DNA. PLoS One. 2015; 10:e0140712. 10.1371/journal.pone.0140712. 26474073 PMC4608804

[42] Struss WJ , Vandekerkhove G , Annala M , Chi KN , Gleave ME , Wyatt A . Detection of circulating tumor DNA in de novo metastatic castrate sensitive prostate cancer. Annals of Oncology. 2018; 29:viii273. 10.1093/annonc/mdy284.005.

[43] Zhen JT , Syed J , Nguyen KA , Leapman MS , Agarwal N , Brierley K , Llor X , Hofstatter E , Shuch B . Genetic testing for hereditary prostate cancer: Current status and limitations. Cancer. 2018; 124:3105–17. 10.1002/cncr.31316. 29669169

[44] Hawkins R . Managing the pre- and post-analytical phases of the total testing process. Ann Lab Med. 2012; 32:5–16. 10.3343/alm.2012.32.1.5. 22259773 PMC3255486

[45] Wickham CL , Sarsfield P , Joyner MV , Jones DB , Ellard S , Wilkins B . Formic acid decalcification of bone marrow trephines degrades DNA: alternative use of EDTA allows the amplification and sequencing of relatively long PCR products. Mol Pathol. 2000; 53:336. 10.1136/mp.53.6.336. 11193054 PMC1186990

[46] Marusyk A , Polyak K . Tumor heterogeneity: causes and consequences. Biochim Biophys Acta. 2010; 1805:105–17. 10.1016/j.bbcan.2009.11.002. 19931353 PMC2814927

[47] Compton CC , Robb JA , Anderson MW , Berry AB , Birdsong GG , Bloom KJ , Branton PA , Crothers JW , Cushman-Vokoun AM , Hicks DG , Khoury JD , Laser J , Marshall CB , et al. Preanalytics and Precision Pathology: Pathology Practices to Ensure Molecular Integrity of Cancer Patient Biospecimens for Precision Medicine. Arch Pathol Lab Med. 2019; 143:1346–63. 10.5858/arpa.2019-0009-SA. 31329478

[48] Bass BP , Engel KB , Greytak SR , Moore HM . A review of preanalytical factors affecting molecular, protein, and morphological analysis of formalin-fixed, paraffin-embedded (FFPE) tissue: how well do you know your FFPE specimen? Arch Pathol Lab Med. 2014; 138:1520–30. 10.5858/arpa.2013-0691-RA. 25357115

[49] Capoluongo E , Ellison G , López-Guerrero JA , Penault-Llorca F , Ligtenberg MJL , Banerjee S , Singer C , Friedman E , Markiefka B , Schirmacher P , Büttner R , van Asperen CJ , Ray-Coquard I , et al. Guidance Statement On BRCA1/2 Tumor Testing in Ovarian Cancer Patients. Semin Oncol. 2017; 44:187–97. 10.1053/j.seminoncol.2017.08.004. 29248130

[50] Einaga N , Yoshida A , Noda H , Suemitsu M , Nakayama Y , Sakurada A , Kawaji Y , Yamaguchi H , Sasaki Y , Tokino T , Esumi M . Assessment of the quality of DNA from various formalin-fixed paraffin-embedded (FFPE) tissues and the use of this DNA for next-generation sequencing (NGS) with no artifactual mutation. PLoS One. 2017; 12:e0176280. 10.1371/journal.pone.0176280. 28498833 PMC5428915

[51] Kokkat TJ , Patel MS , McGarvey D , LiVolsi VA , Baloch ZW . Archived formalin-fixed paraffin-embedded (FFPE) blocks: A valuable underexploited resource for extraction of DNA, RNA, and protein. Biopreserv Biobank. 2013; 11:101–06. 10.1089/bio.2012.0052. 24845430 PMC4077003

[52] Cree IA , Deans Z , Ligtenberg MJ , Normanno N , Edsjö A , Rouleau E , Solé F , Thunnissen E , Timens W , Schuuring E , Dequeker E , Murray S , Dietel M , et al, and European Society of Pathology Task Force on Quality Assurance in Molecular Pathology, and Royal College of Pathologists. Guidance for laboratories performing molecular pathology for cancer patients. J Clin Pathol. 2014; 67:923–31. 10.1136/jclinpath-2014-202404. 25012948 PMC4215286

[53] Gorgannezhad L , Umer M , Islam MN , Nguyen NT , Shiddiky MJA . Circulating tumor DNA and liquid biopsy: opportunities, challenges, and recent advances in detection technologies. Lab Chip. 2018; 18:1174–96. 10.1039/C8LC00100F. 29569666

[54] Neumann MHD , Bender S , Krahn T , Schlange T . ctDNA and CTCs in Liquid Biopsy - Current Status and Where We Need to Progress. Comput Struct Biotechnol J. 2018; 16:190–95. 10.1016/j.csbj.2018.05.002. 29977481 PMC6024152

[55] Elazezy M , Joosse SA . Techniques of using circulating tumor DNA as a liquid biopsy component in cancer management. Comput Struct Biotechnol J. 2018; 16:370–78. 10.1016/j.csbj.2018.10.002. 30364656 PMC6197739

[56] Giannini C , Oelkers MM , Edwards WD , Aubry MC , Muncil MM , Mohamud KH , Sandleback SG , Nowak JM , Bridgeman A , Brown ME , Cheville JC . Maintaining clinical tissue archives and supporting human research: challenges and solutions. Arch Pathol Lab Med. 2011; 135:347–53. 10.5858/2010-0044-SA.1. 21366459

[57] National Accreditation Board for Testing and Calibration Laboratories (NABL) of India. Specific Criteria for Accreditation of Medical Laboratories. 2019. http://13.127.105.244/nabl/index.php?c=publicaccredationdoc&m=index&docType=both&Itemid=199.

[58] Richards S , Aziz N , Bale S , Bick D , Das S , Gastier-Foster J , Grody WW , Hegde M , Lyon E , Spector E , Voelkerding K , Rehm HL , and ACMG Laboratory Quality Assurance Committee. Standards and guidelines for the interpretation of sequence variants: a joint consensus recommendation of the American College of Medical Genetics and Genomics and the Association for Molecular Pathology. Genet Med. 2015; 17:405–24. 10.1038/gim.2015.30. 25741868 PMC4544753

[59] Wagner AH , Walsh B , Mayfield G , Tamborero D , Sonkin D , Krysiak K , Deu-Pons J , Duren RP , Gao J , McMurry J , Patterson S , Del Vecchio Fitz C , Pitel BA , et al, and Variant Interpretation for Cancer Consortium. A harmonized meta-knowledgebase of clinical interpretations of somatic genomic variants in cancer. Nat Genet. 2020; 52:448–57. 10.1038/s41588-020-0603-8. 32246132 PMC7127986

[60] Gradishar W , Johnson K , Brown K , Mundt E , Manley S . Clinical Variant Classification: A Comparison of Public Databases and a Commercial Testing Laboratory. Oncologist. 2017; 22:797–803. 10.1634/theoncologist.2016-0431. 28408614 PMC5507641

[61] PDQ Cancer Genetics Editorial Board. Cancer Genetics Risk Assessment and Counseling (PDQ^®^): Health Professional Version. 2020 May 8. In: PDQ Cancer Information Summaries. Bethesda (MD): National Cancer Institute (US). 2002. https://www.ncbi.nlm.nih.gov/books/NBK65817/. 26389258

[62] Giri VN , Obeid E , Hegarty SE , Gross L , Bealin L , Hyatt C , Fang CY , Leader A . Understanding of multigene test results among males undergoing germline testing for inherited prostate cancer: Implications for genetic counseling. Prostate. 2018; 78:879–88. 10.1002/pros.23535. 29655297 PMC6047906

[63] Abacan M , Alsubaie L , Barlow-Stewart K , Caanen B , Cordier C , Courtney E , Davoine E , Edwards J , Elackatt NJ , Gardiner K , Guan Y , Huang LH , Malmgren CI , et al. The Global State of the Genetic Counseling Profession. Eur J Hum Genet. 2019; 27:183–97. 10.1038/s41431-018-0252-x. 30291341 PMC6336871

[64] Clarke N , Wiechno P , Alekseev B , Sala N , Jones R , Kocak I , Chiuri VE , Jassem J , Fléchon A , Redfern C , Goessl C , Burgents J , Kozarski R , et al. Olaparib combined with abiraterone in patients with metastatic castration-resistant prostate cancer: a randomised, double-blind, placebo-controlled, phase 2 trial. Lancet Oncol. 2018; 19:975–86. 10.1016/S1470-2045(18)30365-6. 29880291

[65] Lynparza 100 mg Film-Coated Tablets - Summary of Product Characteristics (SmPC) - (emc). 2021. https://www.medicines.org.uk/emc/product/9204/smpc.

[66] ^Pr^Lynparza Olaparib 100 mg and 150 mg Film-Coated Tablets. Product Monograph. 2021. https://www.astrazeneca.ca/content/dam/az-ca/downloads/productinformation/lynparza-tablets-product-monograph-en.pdf.

[67] India-List of drugs Approved from SND Division till 31 DEC 2020. 2021. https://cdsco.gov.in/opencms/resources/UploadCDSCOWeb/2018/UploadApprovalMarketingFDC/List%20of%20drugs%20Approved%20from%20SND%20Division%20till%2031%20DEC%202020.pdf.

[68] Giri VN , Gross L , Gomella LG , Hyatt C . How I Do It: Genetic counseling and genetic testing for inherited prostate cancer. Can J Urol. 2016; 23:8247–53. 27085833

[69] Pritchard CC . Genetic Testing for Prostate Cancer in Clinical Practice. JCO Precision Oncology. 2017; 1:1–3. 10.1200/PO.17.00038 35172486

[70] Konstantinopoulos PA , Norquist B , Lacchetti C , Armstrong D , Grisham RN , Goodfellow PJ , Kohn EC , Levine DA , Liu JF , Lu KH , Sparacio D , Annunziata CM . Germline and Somatic Tumor Testing in Epithelial Ovarian Cancer: ASCO Guideline. J Clin Oncol. 2020; 38:1222–45. 10.1200/JCO.19.02960. 31986064 PMC8842911

